# Supplementary material for: Inhibitory control of frontal metastability sets the temporal signature of cognition
Source: eLife. 2022 May 30;11:e63795. doi: 10.7554/eLife.63795 (PMC9200403; doi:10.7554/eLife.63795)
Supplement: Figure 5—source data 1. [file elife-63795-fig5-data1.docx]

| Observable | | **LAT** | | **TAU** | | **Distinct**  **correct effects**  **occur**  **at similar**  **parameter values?** | **Correct effects**  **occur at realistic**  **network dynamics (silence, saturation)?** |
| --- | --- | --- | --- | --- | --- | --- | --- |
| Neuron type | | **Exc.** | **Inh.** | **Exc.** | **Inh.** |  |  |
| Expected  LPFC vs MCC  difference ? | | **YES** | **NO** | **YES** | **YES** |  |  |
| **Parameter** | **g_CAN_** | **↓** | **NO** | **↑** | **NO** | **NO** | **NO** |
|  | **g_AHP_** | **↑** | **↓** | **NO** | **NO** | **YES** | **YES** |
|  | **g_NMDA_** | **NO** | **NO** | **↑** | **↑** | **YES** | **NO** |
|  | **g_GABA-B_** | **NO** | **NO** | **↑** | **↑** | **YES** | **YES** |

**Figure 5 – source data 1**. **Summary of the effects of the main parameters determining TAU and LAT in the network model.**
