# Supplementary material for: Inhibitory control of frontal metastability sets the temporal signature of cognition
Source: eLife. 2022 May 30;11:e63795. doi: 10.7554/eLife.63795 (PMC9200403; doi:10.7554/eLife.63795)
Supplement: Figure 7—source data 1. — Mean firing rates, coefficient of variations (CV), CV2, Lv, and Fano factors in monkey and model data for individual neurons, and averaged them across all neurons in each case. For the model, reported values are averaged over the network and across 100 simulations. [file elife-63795-fig7-data1.docx]

|  | **Frequency (Hz)** | **CV** | **CV2** | **Lv** | **Fano**  **factor** |
| --- | --- | --- | --- | --- | --- |
| **Monkey LPFC** | 4.829 Hz | 1.624 | 1.025 | 1.063 | 0.9976 |
| **Monkey MCC** | 4.995 Hz | 1.614 | 0.9355 | 0.9181 | 0.9975 |
| **Model LPFC** | 2.512 Hz | 1.088 | 0.9169 | 0.8519 | 0.9987 |
| **Model MCC** | 1.144 Hz | 1.78 | 1.025 | 1.103 | 0.9994 |

**Figure 7 – source data 1. Spiking statistics comparison between monkey and model data.** Mean firing rates, coefficient of variations (CV), CV2, Lv, and Fano factors in monkey and model data. for individual neurons, and averaged them across all neurons in each case. For the model, reported values are averaged over the network and across 100 simulations.
