# Supplementary material for: Inhibitory control of frontal metastability sets the temporal signature of cognition
Source: eLife. 2022 May 30;11:e63795. doi: 10.7554/eLife.63795 (PMC9200403; doi:10.7554/eLife.63795)
Supplement: Figure 7—source data 2. — Neurons are divided into two halves – most or least drifting – according to how much their frequency drifts across time to then analyse the spiking activity of each group via HMM (on data from 0 to 600 s). The neural frequency drift averaged across neurons is ~6.6× higher in most drifting vs. least drifting neurons across areas. The average HMM state duration increased by ~1.7× in most vs. least drifting neurons across areas, whereas the ratio of MCC vs. LPFC average state duration across groups was ~5.75×. Thus, neural frequency drift causally increases HMM state durations, but not enough to cause the difference between lateral prefrontal cortex (LPFC) and midcingulate cortex (MCC) average state durations. [file elife-63795-fig7-data2.docx]

| **Average neural frequency drift (Hz)** | LPFC | MCC |
| --- | --- | --- |
| Least drifting neurons | 0.2532 Hz | 0.2219 Hz |
| Most drifting neurons | 1.664 Hz | 1.476 Hz |
| **Average HMM state durations (ms)** | LPFC | MCC |
| Least drifting neurons | 273.75 ms | 1529.25 ms |
| Most drifting neurons | 456 ms | 2754.5 ms |

**Figure 7 – source data 2. Analyzing the causal relationship between neural frequency drift and HMM state durations in monkey spike data.** Neurons are divided into two halves – most or least drifting – according to how much their frequency drifts across time to then analyze the spiking activity of each group via HMM (on data from 0-600s). The neural frequency drift averaged across neurons is ~6.6x higher in most drifting vs. least drifting neurons across areas. The average HMM state duration increased by ~1.7x in most vs. least drifting neurons across areas, whereas the ratio of MCC vs. LPFC average state duration across groups was ~5.75x. Thus, neural frequency drift causally increases HMM state durations, but not enough to cause the difference between LPFC and MCC average state durations.
